# Personal identifiability of user tracking data during observation of 360-degree VR video

**DOI:** 10.1038/s41598-020-74486-y

**Published:** 2020-10-15

**Authors:** Mark Roman Miller, Fernanda Herrera, Hanseul Jun, James A. Landay, Jeremy N. Bailenson

**Affiliations:** Stanford University, Stanford, CA USA

**Keywords:** Computer science, Bone quality and biomechanics, Human behaviour

## Abstract

Virtual reality (VR) is a technology that is gaining traction in the consumer market. With it comes an unprecedented ability to track body motions. These body motions are diagnostic of personal identity, medical conditions, and mental states. Previous work has focused on the identifiability of body motions in idealized situations in which some action is chosen by the study designer. In contrast, our work tests the identifiability of users under typical VR viewing circumstances, with no specially designed identifying task. Out of a pool of 511 participants, the system identifies 95% of users correctly when trained on less than 5 min of tracking data per person. We argue these results show nonverbal data should be understood by the public and by researchers as personally identifying data.

## Introduction

Virtual reality, the use of computational technology to create a simulated environment, has spiked in use in recent years^1^. In order to render the virtual world from the viewpoint of the user, the user’s position must be calculated and tracked. All VR systems measure head orientation, most measure head position, and many measure hand orientation and position^2^. While less common, some can track feet, chest, and even elbows and knees to increase immersion^3^. This data is a problem for users concerned about privacy. The tracking data VR provides can be identifying.

In contrast to previous work, which has focused on designing VR tasks to identify or authenticate users^4–6^, we begin with a task that was not designed for identification. In fact, the tracking data we use is from a study^7^ designed with the intention examining the associations between motion, self-report emotion data, and video content.

Moreover, the current work is unique in that it uses a very large (over 500) and diverse sample primarily from outside a university. Both features of the sample are relevant theoretically. First, identification in small samples likely overestimates the diagnosticity of certain features given the lack of overlap in body size and other sources of variance. Second, different people likely have different types of body movements. For example, the study has over 60 participants over the age of 55, and the movement of this group is likely different from a typical college student.

If tracking data is by nature identifying, there are important implications for privacy as VR becomes more popular. The most pressing class of issues falls under the process of de-identifying data. It is standard practice in releasing research datasets or sharing VR data to remove any information that can identify participants or users. In both the privacy policy of Oculus and HTC, makers of two of the most popular VR headsets in 2020, the companies are permitted to share any de-identified data. If the tracking data is shared according to rules for de-identified data, then regardless of what is promised in principle, in practice taking one’s name off a dataset accomplishes very little.

The second class of threats is broadly concerned with an improved ability to link VR sessions together. Information that was previously scattered and separate is now able to be joined by a “motion signature.” In connecting some tracking data to a name, for example, now tracking data in many other places are attached to the same name. This increases the effectiveness of threats based upon inference of protected health information from tracking data (for examples, see Related Work).

A third class of threats stems from "private browsing". In principle, there is a way to enter a "private browsing mode" in a web browser. While it may be difficult and require many tools hiding many layers of information, it is possible. With accurate VR tracking data, a "private browsing mode" is in principle impossible.

Using only the position tracking data, we find that even with more than 500 participants to choose from, a simple machine learning model can identify participants from less than 5 min of tracking data at above 95% accuracy. Therefore, we contribute data suggesting typical VR experiences produce identifying data. Moreover, by examining different types of models and different feature sets, we shed light on the possible mechanism behind identification, which also suggest at strategies to prevent abuse.

In this paper, first two threads of related work are reviewed: methods of identifying users through tracking data, and concerns about VR privacy. Second, the experimental setup and data collection processes are reported. Finally, the results of each identification task are reported, and results are discussed.

## Related work

Previous work has used tracking data to identify users, but identification is framed positively, often as a tool for authentication. The ongoing adoption of VR has raised concerns about VR tracking data as a privacy concern.

### Privacy issues of VR tracking data

VR tracking data, as a measure of body pose and motion, is a surprisingly powerful source of information. Associations have been made between kinds of body motion and creativity^8^, learning^9^, and other behavioral outcomes^10^.

Furthermore, behaviors captured in tracking data can be associated with medical conditions such as ADHD^11^, autism^12^, and PTSD^13^. There is also growing literature in the use of tracking data to diagnose dementia^14–16^.

From these examples, a pattern emerges. Each of these tests are an everyday scene in which the experimenter measures some behavior known to be indicative of some medical condition (e.g., distraction, attention to faces, duration in motion planning). The ability to find these associations merely from tracking data has brought researchers’ attentions to privacy in VR^17^.

Hosfelt^18^ discusses immersive media ethics and notes the power of collecting involuntary nonverbal interactions. Responses that we expect to be private may be quickly detected by algorithms while we are unaware. Vitak et al.^19^ grounds the discussion of VR privacy within a larger context of networked privacy. As with many privacy concerns, the question hinges on whether the gains to the user outweigh the concerns.

### Identifying users by motion from a head-mounted display

Previous work on identifying users of head-mounted displays have used different types of biometric measurements. The most common is head rotation and acceleration through inertial measurement units in the device^4,6,20,21^. With the rise in popularity of VR headsets, position and orientation data of the headset and hand controllers have become more common data sources^5,22^.

The purpose of determining the user’s identity may be for authentication or identification. We follow the distinction Rogers et al.^6^ make between authentication and identification. Authentication requires strong positive evidence identifying a single user from any other user of a system, and usually leads to elevated privileges (e.g., access to sensitive data, or ability to run more powerful commands). Identification, on the other hand, is the matching of a user within a predefined set of an arbitrary (but finite) number of users. For example, identification might be used to automatically apply user preferences or create personalized advertisements.

Previous work has focused on finding a task that can identify users. For example, users can nod their head in response to an audio clip, creating a “motion password”^20^. There has been interest in making these authentication tasks less intrusive. For example, Kupin et al.^22^ track users as they make a throwing motion, and Mustafa et al.^4^ track as participants walk around a room. However, little work has been done in determining identity from data gathered from an experience designed with no intention of identification.

## Methods

Participants watched 360-degree videos and answered questionnaires in VR. The tracking data was summarized and processed as input for three machine learning algorithms. The study was approved by the Stanford University IRB under protocol number 43879, and all methods were performed in accordance with these guidelines.

### Participants

**Figure 1:**
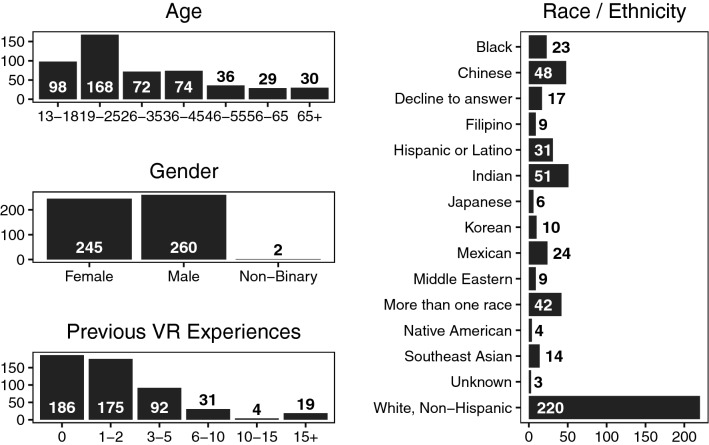
Histograms of demographic information (age, gender, number of previous VR experiences, and race and ethnicity) of study participants.

A total of 548 participants were recruited to participate in the study. Of these, 10 did not permit their tracking data to be used as part of the analysis, and 27 did not finish the five videos for one of various reasons: 7 ended early to save time, often because they had a reservation for another museum activity, 6 did not specify a reason when asked, 5 could not read the survey text, 4 ended due to simulator sickness or eyestrain, 3 ended due to content (2 did not like the crowds and 1 did not like the animals) and 2 ended due to uncomfortable headset fits. In total, 511 participants completed the experiment. There were 378 at the museum and 133 from campus. Demographic information is given in Fig. 1. Ages are given in ranges rather than raw values because participants selected were not able to type in their age but rather select one of the seven options. Race and Ethnicity is given using categories adapted from the 2010 United States Census. The full text of each question, as well as its answer options, can be found in the study’s online repository^23^.

All adult participants read and signed an IRB-approved consent form. To run participants younger than 18, child assent and parental consent were required. If participants at the museum opted out of participating in research, they still had the option to view the 360 videos without having their data collected.

### Apparatus

**Figure 2:**
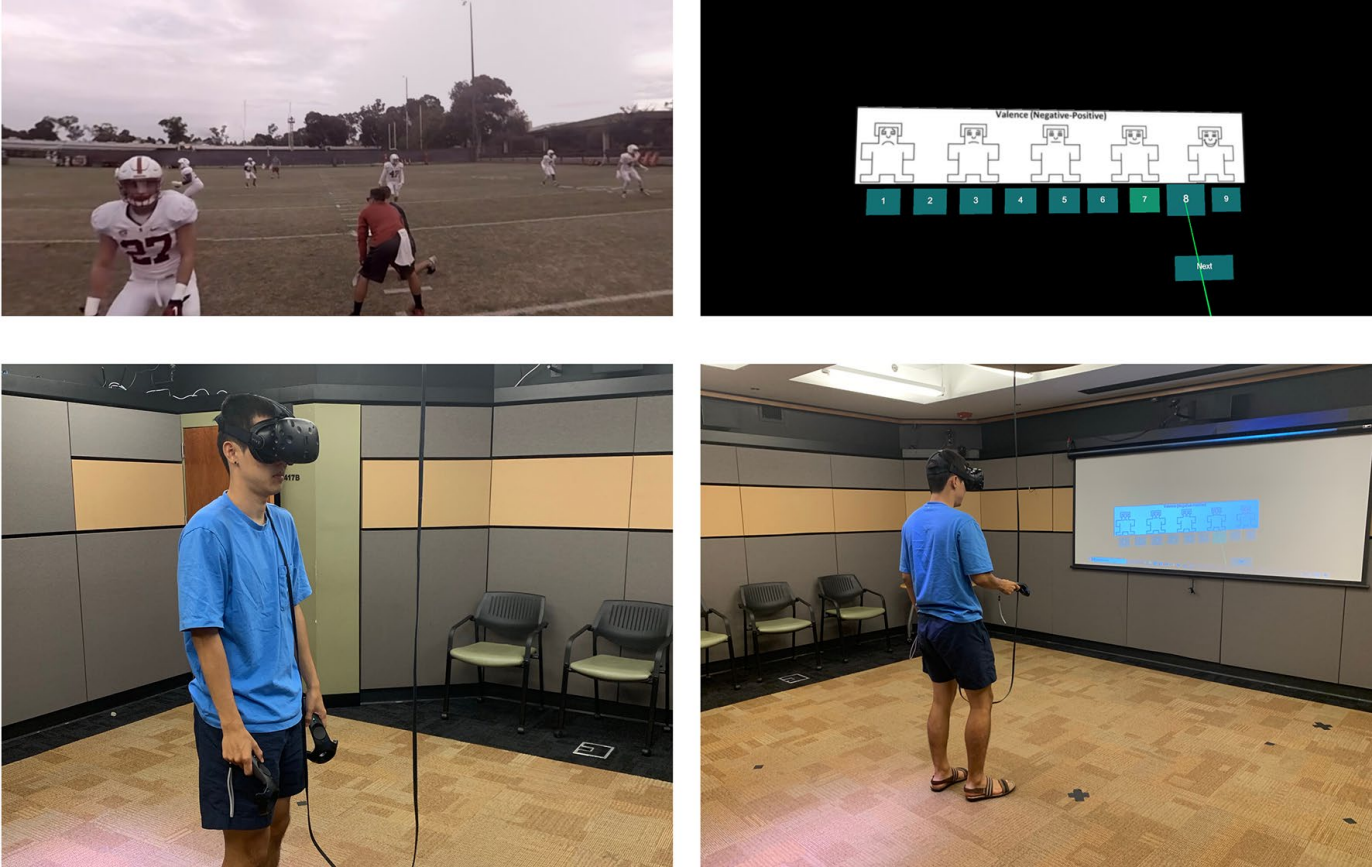
Experimental setup. Top left: a screenshot of one of the 360-degree videos. Top right: a screenshot of the VR questionnaire. Bottom left and right: photograph of co-author demonstrating the process of watching video and answering survey during the experiment.

Virtual reality content was displayed using the HTC Vive virtual reality headset and controllers^24^. The experimental space on campus is shown in Fig. 2.

### Stimuli

The virtual reality content in question consisted of five 360-degree videos, each 20 s long, randomly selected from a set of 80 videos. The videos were gathered to range in valence (positive versus negative) and arousal (calm versus exciting). Permission to display videos and allow researcher reuse was obtained for each video from the original videographer, and all videos are available through the study repository^23^.

All videos were stored locally on the computer connected to the VR headset. The framerates of the videos varied from 23.97 to 120, with 8 videos with fewer than 29.97fps, 66 videos with framerates between 29.97 and 30fps, and 6 videos with more than 30 fps. The resolution of the videos ranged from 1920 by 960 pixels up to 4096 by 2048 pixels, with 39 videos at the top end of this range.

Given 360-degree video offers viewing in any direction, it is also important to consider the effect of the content itself on motion. If the content causes many people to move in similar ways, identification by motion may be more difficult. Two features that may cause this are spoken narrations and the strength of the video’s focal point. There were no videos that had an external narrator telling the viewer where to look, but there were sixteen videos in which a human voice could be heard, and of those, nine where specific words and specific speakers could be made out. The focal point of videos were strongest in videos of a single animal (e.g., elephant, baboon, giraffe) that paced around the camera, and focal points were weakest in videos of nature scenes with trees visible in all directions. Videos with scene changes, camera rotation, or jerky camera motion were excluded beforehand. Only one video involved motion of the camera, and the camera was moving at a slow constant velocity.

### Experimental design and protocol

Researchers recruited participants at two locations: a technology museum based in a large city, and students from a university. Researchers obtained informed consent from all participants over 18 using a consent form. For participants younger than 18, a parent or legal guardian gave informed consent through a parental consent form and the participant gave assent through an assent form. After consent was obtained, the researcher set up the participant with the VR headset and hand controllers, confirmed the fit and comfort of the headset, and began the virtual reality application.

Within the virtual reality application, participants answered a demographic questionnaire. When those questions were completed, the program randomly selected five videos. For each of the videos, the program displayed the video to the participant and then prompted the participant with questions about valence, arousal^25^, presence, simulator sickness, and desire to share the content. The full text of questionnaires are available in the study repository^23^. The substantive results relating to content from these questionnaires have been investigated in separate publication^7^ focused on VR content and use. Once the participant answered these questions, the program loaded another video, displayed it, and prompted with another round of questions. This repeated five times for the 378 participants at the museum and eight times for the 133 participants on campus. For the analyses reported in the current paper, any trial from the university past the fifth was ignored to ensure the datasets were consistent.

When the final video had been displayed, the researcher helped the participant out of the VR headset, answered any questions, and debriefed the participant about the experiment.

### Data processing and statistical analysis

Raw tracking data was collected at 90 Hz. The data collection rate was stable, with only 0.05% of frames coming more than 30 ms after the previous frame. In each frame, the timestamp and button presses were recorded, along with the 6DOF (i.e., position and rotation) of the headset, left hand controller, and right hand controller.

The three positional dimensions are denoted as X, Y, and Z. In the conventions of Unity, the game engine the virtual reality experience was developed in, the Y axis is vertical, the Z axis is forward–backward, and the X axis is left–right. The three rotational dimensions are yaw (rotations about the vertical axis), pitch (rotation tilting upwards or downwards, about the left–right or X axis) and roll (rotation around the forward–backward or Z axis). Some of these measures have straightforward spatial meanings, e.g. the y-axis captures how high the tracked object (headset or hand controller) is from the ground, and headset yaw indicates the horizontal direction the participant is looking.

To summarize the data into *samples*, multi-dimensional vectors appropriate for the machine learning algorithms, the tracking data was binned into 1-s chunks. A value was calculated for each combination of summary statistic (maximum, minimum, median, mean, and standard deviation), body part (head, left hand, right hand), and dimension (x, y, z, yaw, pitch, and roll). This resulted in a 90-dimensional vector for each second of each video session. The process of dividing variable length data into chunks and summarizing chunks is a process performed in previous methods^5^ of body motion and identification. In total, there were 61,121 samples in the video task, and 115,093 samples in the survey task. Breaking down the number of samples per person, there was an average of 345 samples, a standard deviation of 60.5 samples, and a range from 214 to 663.

These samples were collected across ten *sessions* for each participant, which are delineated by the beginning of each of the videos (of which there were five) and the task being performed (either watching the video, or answering the survey that followed the corresponding video). Breaking down the number of samples per session, there was an average of 34.5 samples, a standard deviation of 16 samples, and a range from 13 to 178 samples.

#### Performance metric

Accuracy is evaluated upon predictions given per-session rather than per-sample (i.e., a single prediction for each session of time watching one video, or answering one video’s survey, rather than a prediction per second). This is done by collecting the predictions of each second of the task as votes and designating the session’s prediction as the prediction with the most votes.

Following Pfeuffer et al.^5^, classification accuracy, (i.e., the percentage of correct predictions to total predictions made) is the chosen metric of performance. While related papers have used equal error rate^4,20^ or balanced accuracy rate^6^, they are authentication tasks where risks are asymmetric. A false negative is an annoyance for the user, but a false positive compromises the security of the system. In identification tasks such as our work, we assume the cost of error is independent of the true and predicted classes. Furthermore, there are an equal number of examples in each class, as predictions are evaluated per-session rather than per-second, so a naïve most-common-entry classifier cannot perform better than chance (1/511, or about 0.2%). Therefore, classification accuracy provides an unbiased, easily understandable measure of performance.

The accuracy values reported in this paper, unless specified otherwise, come from the average of twenty Monte Carlo cross-validations, stratified by participant and task. For each participant and task, one session’s data is randomly chosen and left out of the training process. Once the model has been trained, it is tested against this left-out data. Then, the old model is thrown out, a new training and testing split is made, and a new model is evaluated.

#### Algorithms

Three machine learning models were piloted: k-nearest-neighbors (kNN)^26^, random forest^27^, and gradient boosting machine^28^ (GBM). All methods were run in R version 3.5.3, with ‘class’ version 7.3-15, ‘randomForest’ version 4.6-14, and ‘gbm’ version 2.1.5.

While previous work has used support vector machines (SVMs) to identify users, SVMs do not directly support multiclass classification. The commonly used R library for SVM, ‘e1071′, works around this by training *O(n*^2^*)* one-to-one classifiers, one for each pair of classes. Unfortunately, this implied training over 100,000 SVM classifiers, which was computationally intractable on available machines.

The choices of kNN, random forest, and GBM, were motivated in different ways. Nearest-neighbor approximation is a robust algorithm that can be easily understood with little statistical background^26^. The random forest method has seen success in previous body motion identification tasks^5,6^. GBM is similar to random forest in that both use decision trees, but instead of creating many trees that vote on predictions called an *ensemble* model, each tree is trained to reduce subsequent error in a process called *boosting*. GBM can create more accurate predictions than random forests but often require many more trees to do so.

Each algorithm accepted a 90-element motion summary vector as input, and it output a classification prediction representing one of the 511 participants. For kNN, each variable is normalized to mean zero and variance one before computing the Euclidean distances between points. The RandomForest parameters were set to R defaults, except that 100 trees were trained. GBM was run with 20 trees, with an interaction depth of 10 and a minimum of 10 observations per node. The number of trees in the GBM model is small because of the computational intensity of classifying between 511 classes. This likely reduced the algorithm’s performance.

Once the algorithms are compared, we will select the most successful of them to continue further analysis.

## Results

Using the VR tracking data, models are evaluated to determine how and when identification can take place.

### Identity prediction

**Figure 3:**
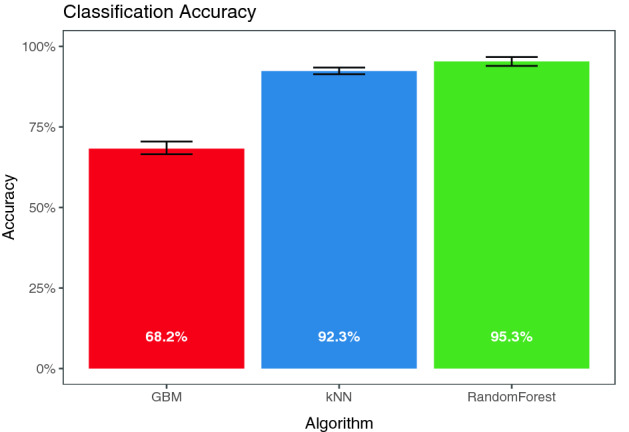
Accuracy of all methods when trained and tested on both kinds of tasks. Error bars show the range of all 20 Monte-Carlo cross-validations.

The question that must be asked first is whether VR produces identifying information. Classification accuracy is reported below. All algorithms performed much better than chance, but Random Forest and kNN performed best (Fig. 3). These results clearly demonstrate identifying data is produced when recording motion during a VR experience.

There are random variations in accuracy due to the stochastic nature of the Monte Carlo cross-validation method and two of the algorithms (random forest and GBM). In order to discount the possibility these results were achieved by variation due to the algorithm or cross-validation, we use two-sided t-tests to compare the distribution of the accuracy of the 20 cross-validations against a null hypothesis of accuracy being chance (1/511, about 0.2%). Each of the three algorithms performed significantly better than chance: GBM (t(19) = 309, *p* < 0.001, CI = [0.678, 0.687]), kNN (t(19) = 780, *p* < 0.001, CI = [0.921, 0.926]), and Random Forest (t(19) = 650, *p* < 0.001, CI = [0.950, 0.956]).

### Identification across tasks

**Figure 4:**
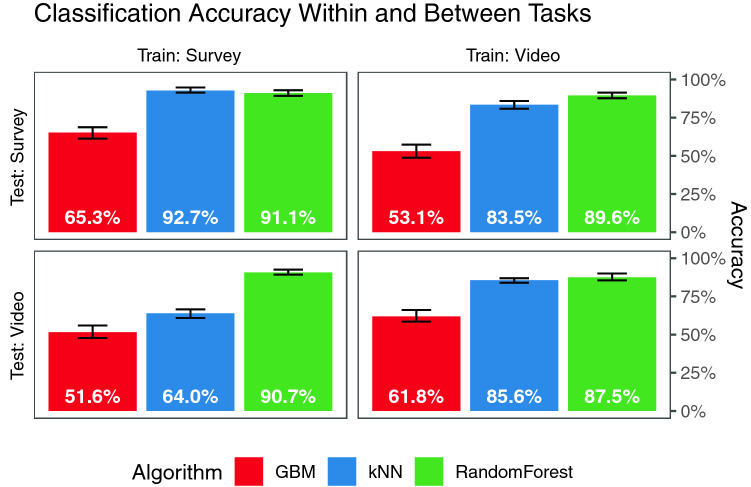
Accuracy within and between tasks. Error bars show the range of all 20 Monte-Carlo cross-validations.

If tracking data produces *robust* identifying information, then identification should occur across multiple tasks. To this end, we investigate participant identification within both tasks in the study, a video-observing task and a questionnaire-answering task. Classification accuracy is given in Fig. 4. In both the survey task (top left) and video task (bottom right) high levels of accuracy are reached. Therefore, identifying information is part of tracking data in both the survey and video tasks.

Second, is the identity learned in one task transferable to another? This is answered by Fig. 4, top right and bottom left. In these cases, a model is trained upon data collected from one task (e.g. survey) and tested upon data collected during another task (e.g. video watching). The algorithms are still accurate.

### Comparison of algorithms

Random forest is the most accurate algorithm among the three in both the condition with all data and three of the four combinations of training and testing tasks. This is evidenced by the significance of an ANOVA predicting accuracy of each of the sixty cross-validations based upon algorithm, F(2, 57) = 7903, *p* < 0.001. Tukey’s HSD post-hoc tests show RandomForest is more accurate than either GBM (*p* < 0.001) or kNN (*p* < 0.001), and that kNN is more accurate than GBM (*p* < 0.001). Note that significant values here mean that the differences are unlikely to be random variation in the running of the algorithm or the selection of cross-validation. Significance here should not be interpreted as empirical evidence that one algorithm performs better than another on this task in general.

Because random forest tended to perform best, we focus on the random forest models in the following research questions.

### Feature importance for prediction

**Figure 5:**
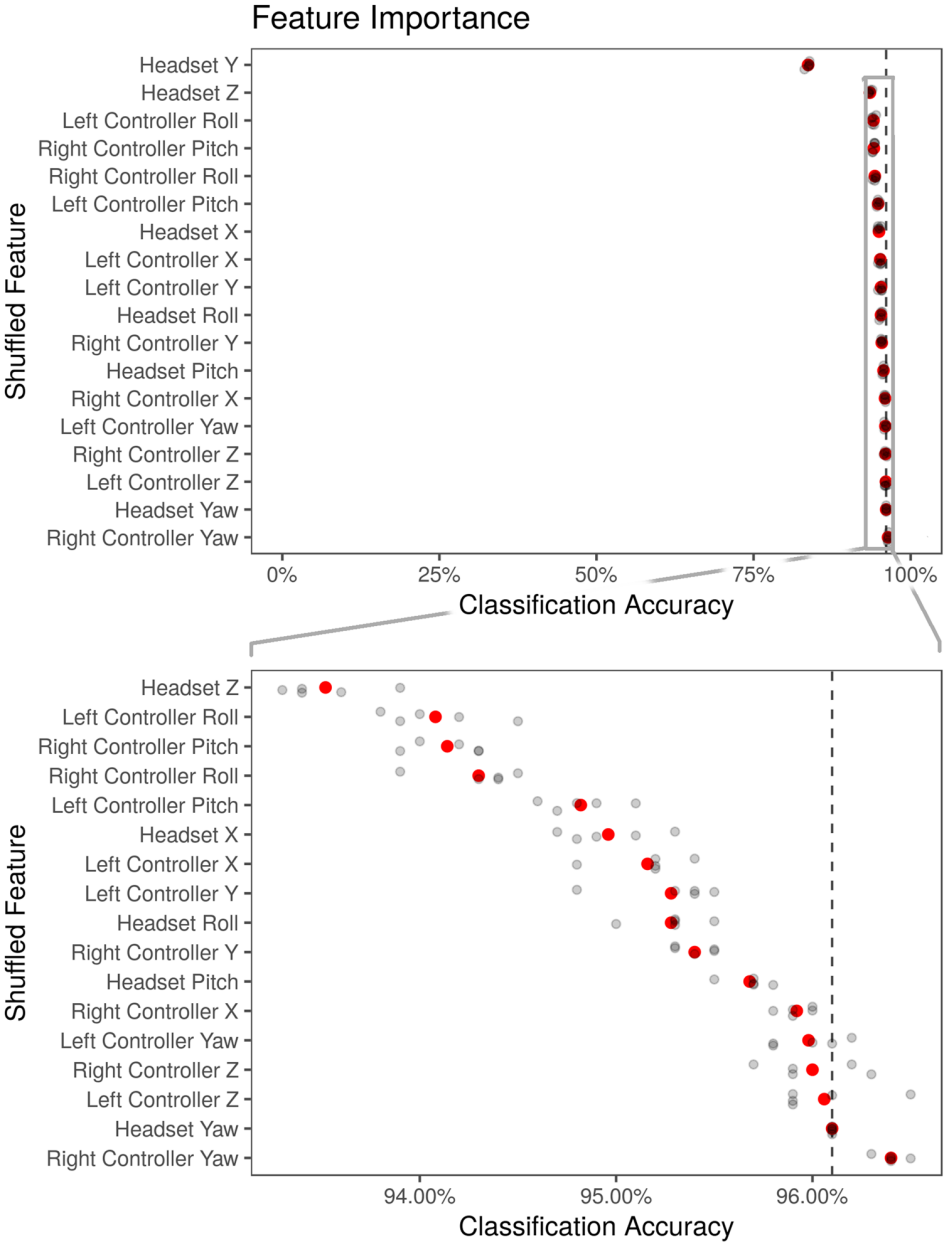
Feature importance displayed as Mean Decease in Accuracy. Black dashed line represents accuracy upon the unshuffled data. Bottom panel is a magnified section of the top panel.

Knowing what features the model makes its decisions upon can provide insight into what makes motion data identifying. A measure of feature importance in random forests is Mean Decrease in Accuracy. In this method, all observations in a predictor variable are shuffled, breaking any relationship between the predictor variable and its outcome. This usually results in a decrease in accuracy, with a larger decrease for variables that the random forest relies upon more often. This decrease in accuracy is plotted in Fig. 5. By far, the most important feature is Headset Y, with an accuracy decrease of about 10 percentage points. Even though that is the largest drop, the model is still correct 82% of the time.

The Y position of the headset roughly corresponds to the participant’s height. Other variables that may influence it are the participant’s posture and fit of the headset. The vertical position of the headset is robust to the common kinds of motion in this VR experience, while also varying much between participants. These two qualities make height a very good predictive variable.

The remaining features have small decreases in accuracy but form a consistent ranking in importance. To illustrate this, a zoomed-in version of the top panel of Fig. 5 is displayed in the bottom panel of Fig. 5. The next most important features are Headset Z (head forward/backward) and the roll and pitch of the hand controllers.

Headset Z roughly corresponds to position forward or backward in the virtual environment. While we expect much of this feature to be determined by where they happened to stand during the VR experience, some of it may be a result of the positioning of VR content. During the time in between sessions, participants answered a questionnaire on the emotional content in the video. This questionnaire was displayed in the same location in VR each time. Depending upon headset fit and eyesight, perhaps participants would step towards the questionnaire to see it more clearly or step away to see more of it at a time.

The next four features, left and right controller roll and pitch, correspond roughly to how participants place their arms and hold the controllers while at rest. Some participants had their arms down by their sides, but some were pointed outward, or had arms crossed. If crossed, were they right-over-left, or left-over right? Participants tended to be consistent with their comfortable resting pose between videos.

Is the model merely learning where someone happens to be standing? Most participants did not walk around the space, so their location horizontally (XZ) would be stable and identifiable. If so, then what is being identified is the VR session, not the participant. This concern is corroborated by the fact that the second most predictive feature is Z Position of the headset, which does not have an easy interpretation.

One method to address this concern is to toss out all horizontal position data completely. When training a separate random forest model upon data with all X and Z position information removed, identification was still 92.5% accurate. While it is less than the original 95.3%, we do not consider it a difference enough to change the narrative of these results.

### Training set size and accuracy

How much data is necessary in order to identify a person? To explore this question, the model was trained on varying amounts of tracking data. To review, each participant saw five videos and answered survey questions after each one. Therefore, each participant had ten sessions to train upon. To keep data balanced, the training set could be 1 video task and 1 survey task per participant (2 sessions total, 20% of all data); 2 each of video and survey tasks (4 sessions total, 40%); 3 each, 6 sessions total (60%); or the size of the original training set of 4 each (8 total, 80%).

**Figure 6:**
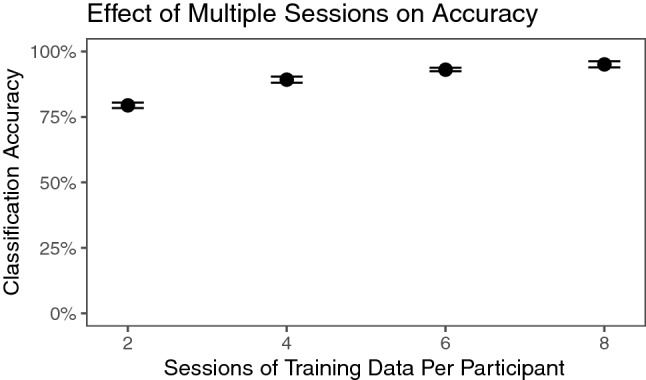
Accuracy given different amounts of training data. Error bars represent the range of accuracies in 20 Monte-Carlo cross-validations.

Results are plotted in Fig. 6. Even with one survey task and one video task, the model can predict identity over 75% of the time. Including each additional video increases accuracy. An ANOVA investigating the relationship among 2, 4, 6, and 8 training sessions per participant among 20 cross-validations per condition showed a significant effect of training set size on accuracy, F(3, 77) = 3078; *p* < 0.001. Tukey’s HSD with Bonferroni correction showed significant differences between all groups, all with *p* < 0.001.

### Identification in 3DOF headsets

Other headsets currently available track only in three rotational dimensions: yaw, pitch, and roll of the user’s head. Considering all three of those dimensions are in the weaker half of feature predictions, it is worth investigating whether a participant still be identified upon this tracking data. When a random forest model was trained upon data consisting only of head yaw, pitch, and roll, participants were identified 19.5% of the time. This is large drop from 95.3% on all data, but participants are still identified 97 times more often than chance.

### Participant set size and accuracy

**Figure 7:**
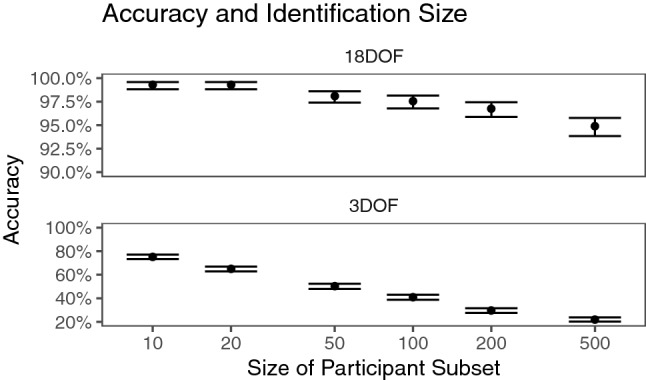
Accuracy and identification size. Translucent dots represent samples, with size specified by x-axis. Intervals represent 95% CI over the binomial success rate. Note the difference in scales between the top and bottom panels: 18DOF (top) uses positional and rotational data of head and hands and provides more features to train on than 3DOF (bottom) does.

In contrast to other identification studies, which usually have had below 30 participants^4,6,21^ or at most 100^20^, this study included 511 participants. Intuitively, it is more difficult to identify someone from a group of 500 than a group of 20 or 100, so it is worth comparing across different size subsets of the data. Figure 7 compares accuracy among several participant subset sizes.

Identification is easiest in smallest groups. In addition, tracking is easier with more data. It appears that accuracy drops more in the 3DOF identification case than the 18DOF case.

## Discussion

VR tracking data produces information that can identify a user out of a pool of 511 people with an accuracy of 95.3%. This accuracy is still high (89–91%) when training occurs on one task and testing with another type of task. The most important features the model uses to make decisions include headset Y (height, posture), headset Z (distance from the VR content, headset fit affecting comfortable focal length), and controller pitch and roll (placement of hand controllers when at rest).

The task being performed in VR will affect the importance of the variables. For example, if participants were not watching a 360-degree video but instead swinging a virtual baseball bat, controller roll would not be consistent, and it would likely not be an identifying measure. However, other features like arm length could be learned from the tracking data.

Because the algorithm is trained upon chunks of one second of motion data, the algorithm must rely upon features visible within one-second chunks of positional data. As evidenced by the feature importance figure, these features are static, e.g. height, arm length, how controllers are held, as opposed to dynamic, e.g. head rotation speed, jerky hand movement, or some idiosyncratic motion pattern when shifting weight. When the training data is reduced, there is still a sizable pool of data from which these static features can be drawn. Even when a model is trained on only one survey task and one video task per person, reducing the total training data time from an average of 276 s to an average of 69 s, classification accuracy only drops from 95% to 75%.

Merely collecting tracking data of an interaction in VR—even if that interaction is not designed to be identifying—is enough to identify users with alarming accuracy. We propose the greater challenge is not to design identifying interactions but rather to design *non-identifying* interactions.

Tracking fewer channels of data may help with this process, as shown above. In addition, displacing positional data by some amount may allow one person to reliably appear as the height of another person. In putting any of these methods into practice, though, one must consider the type of threat that one is protecting against.

## Limitations and future work

A limitation to note is that each participant’s data was collected all in the same day, often within the span of about 10 min and never more than 30 min total. At no point in the process was the headset taken off, the hand controllers removed, or the virtual environment re-loaded. Therefore, some features may not only be capturing similarities between participants but merely between sessions.

To address this limitation, future work can extend this finding using velocity, acceleration, and rotation data. Previous work^4^ has found the use of this kind of tracking data to be feasible.

A limitation of this work is that both tasks captured standing users with little motion. It is difficult to expect these features to be identifying in a VR task that involves a lot of motion, for example, VR tennis. The generalizability beyond the tasks demonstrated here should be tested.

One drawback of this work and most previous work is that the positional data is summarized, reducing dimensionality at the cost of losing information. Learning from raw positional time series data (rather than summary statistics) may produce more robust identifying features. Other branches of human behavior understanding have found success using neural networks^29,30^. To our knowledge, this has not been attempted with VR identification and may be a fruitful avenue to explore.

Furthermore, future work can also explore the feasibility of inferring gender, age, or VR experience based upon tracking data. Then, the model would be not merely matching data but building a user’s profile.

Finally, and most importantly, methods of designing for privacy in VR should continue to be explored. There are many avenues to protect this kind of data, including policy, user behavior, industry guidelines, and others.

With the rise of virtual reality, body tracking data has never been more accurate and more plentiful. There are many good uses of this tracking data, but it can also be abused. This work suggests that tracking data during an everyday VR experience is an effective identifier even in large samples. We encourage the research community to explore methods to protect VR tracking data.

## Data Availability

The dataset of 360-degree VR video is available at https://github.com/vhilab/psych-360. The tracking data generated by participants is available under reasonable request.
